# Prolonged hypereosinophilia associated with dupilumab for atopic dermatitis: A case series and review of literature

**DOI:** 10.1016/j.jdcr.2024.08.003

**Published:** 2024-08-29

**Authors:** Gabriela Fonseca, Sivani Reddy, Tiffany Mayo

**Affiliations:** a University of Alabama at Birmingham Marnix E. Heersink School of Medicine, Birmingham, Alabama; b Department of Dermatology, University of Alabama at Birmingham, Birmingham, Alabama

**Keywords:** atopic dermatitis, dupilumab, eczema, eosinophilia, hypereosinophilia, side effects, skin of color

## Introduction

Atopic dermatitis is a chronic, pruritic immune mediated inflammatory dermatosis characterized by a T helper 2 cell immune response.^1^ It has been treated with topical corticosteroids, oral immunosuppressants, and light phototherapy.^2^ However, dupilumab has been a breakthrough medication in treating atopic dermatitis. Dupilumab is a human monoclonal antibody that blocks interleukin 4 (IL-4) and interleukin 13 (IL-13) and has been shown to be safe and helpful in treating a variety of conditions.^1^ Blockade of IL-4 and IL-13 is effective in reducing the T helper 2 response, likely explaining the mechanism of action in treating atopic dermatitis.^3^ Herein we describe 2 patients with chronic atopic dermatitis who were started on dupilumab and experienced hypereosinophilia, with lack of improvement in atopic dermatitis. Given significant eosinophilia, an extensive workup for malignancy and other potential causes of disease was performed. Ultimately, dupilumab was discontinued and eosinophilia trended toward normal. We conclude with a discussion on recent literature and a potential explanation of eosinophilia associated with dupilumab.

## Case report

### Case 1

**Fig 1:**
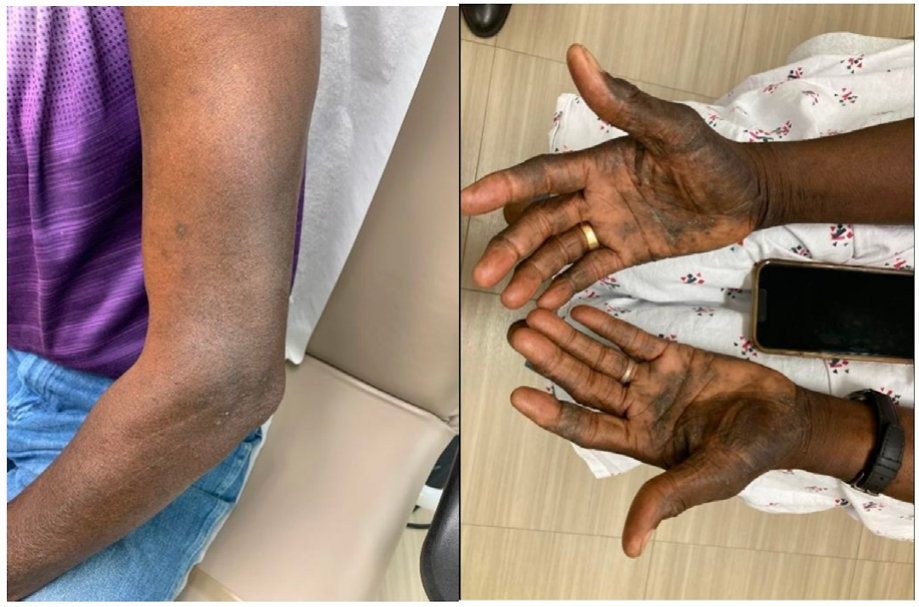
Case 1 patient with hyperpigmented, lichenified plaques on hands and arms consistent with atopic dermatitis.

A 74-year-old African American man presented in August of 2023 with persistent atopic dermatitis despite starting dupilumab 7 months prior. He had seen an outside provider who had diagnosed him based on biopsy and started this medication. In addition, he noted body aches, weight loss, and fatigue that started after initiation of dupilumab (Fig 1). Dupilumab was discontinued and laboratory work revealed an eosinophil count of 47% (reference range 0%-5%, conversion 3948 cells/mm^3^). Biopsy was performed to rule out other causes of eczematous dermatitis, confirming his atopic dermatitis diagnosis. Of note, there was no travel or a medication history. A stool for ova and parasite was not performed. He was referred to hematology/oncology (Hem/Onc) with concern for possible malignancy. Hem/Onc diagnosed him with hypereosinophilic syndrome and referred him to pulmonology, with possible consideration of a bone marrow biopsy. Of note, his eosinophil count had decreased to 31% (3813 cells/mm^3^) in 7 weeks after discontinuing dupilumab. Pulmonology found no respiratory involvement. It was suspected dupilumab was the cause of the patient’s eosinophilia. Without additional intervention, eosinophilia decreased to 16% (804 cells/mm^3^) and musculoskeletal pain and fatigue resolved in December of 2023, 18 weeks after stopping the medication. The patient was started on upadacitinib with notable improvement in atopic dermatitis.

### Case 2

**Fig 2:**
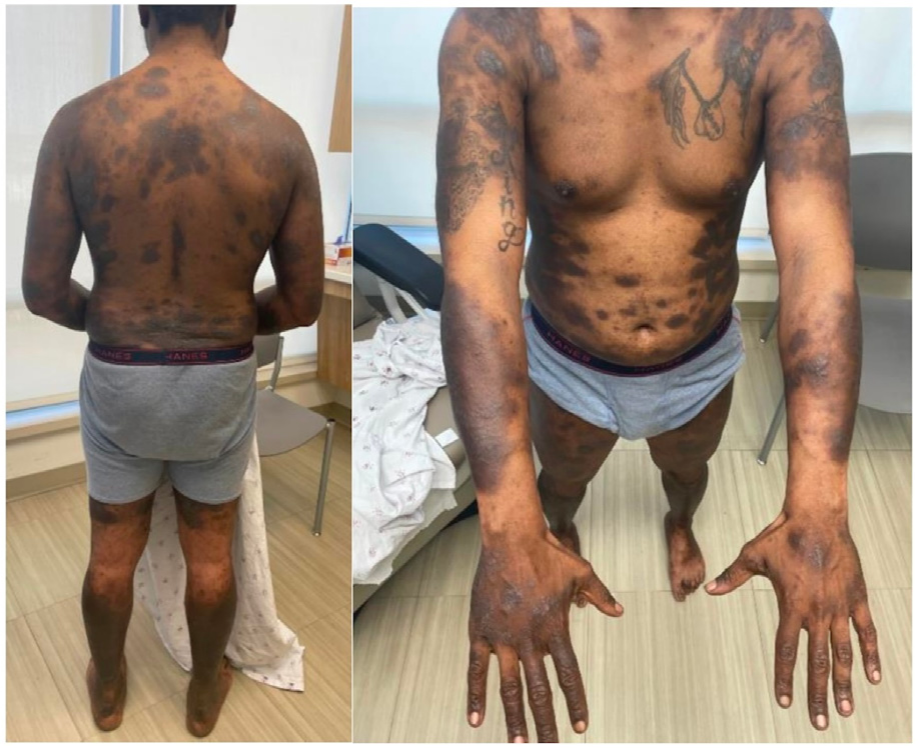
Case 2 patient with hyperpigmented, lichenified plaques on chest, back, arms, and legs.

A 30-year-old African American man presented in August of 2023 with widespread atopic dermatitis (Fig 2), despite having started dupilumab 6 months earlier by an outside provider. There is no record that a biopsy was done at that time. Dupilumab was discontinued and the patient was biopsied to verify his diagnosis, confirming spongiotic dermatitis. There was no travel or medication history. A stool for ova and parasite was not performed. Laboratory tests were notable for eosinophilia of 32% (4675 cells/mm^3^). Patient was referred to Hem/Onc for evaluation of possible hypereosinophilic syndrome. Seven weeks after discontinuing dupilumab, eosinophils had trended down to 13% (1352 cells/mm^3^). Hem/Onc noted eosinophilia was most likely secondary or reactive, but wanted to rule out underlying eosinophilic leukemia or clonal disorder. Bone marrow biopsy was performed with no notable abnormalities. In April 2024, his eosinophils trended down to 8% (950 cells/mm^3^), 18 weeks after discontinuation of dupilumab. The patient was started on upadacitinib and has been doing well.

## Discussion

Although dupilumab is considered a safe medication and very helpful in treating atopic dermatitis, some adverse side effects include dermatitis, psoriasis, arthralgias, alopecia areata, and cutaneous T cell lymphoma.^4^ Eosinophilia (Absolute eosinophil count > 500/mmc) has recently been described as an adverse effect of dupilumab, and is detailed as transient and self-resolving in the context of treating asthma, atopic dermatitis, and chronic rhinosinusitis.^5^^,^^6^ It rarely had any clinical symptoms. Moreover, these reports of eosinophilia upon dupilumab initiation were largely associated with a history of low grade baseline eosinophilia before starting dupilumab and more frequent in patients with concomitant asthma or rhinitis.^4^^,^^7^ For example, in the study by Faiz et al,^7^ eosinophilia at follow-up visits with patients on dupilumab was associated with prior eosinophilia at baseline (64.2% in patients on dupilumab and with baseline eosinophilia vs 13.5% in patients who had not presented with eosinophilia originally), asthma (81.4% vs 49.3%), and allergic rhinitis (67.8% vs 50%). This shows eosinophilia due to dupilumab is also associated with a prior history of eosinophilia, asthma, and rhinitis.

Faiz et al^7^ found that out of 28 patients with hypereosinophilia (>1500 cells/mm^3^), only 5 patients required medication discontinuation because of persistent hypereosinophilia without another etiology. Oral steroids and other medications were started for these patients, which ultimately led to their eosinophils normalizing. Only with further medications did their elevated laboratory tests subsequently resolve.^7^ Persistent, symptomatic hypereosinophilia had been previously seen in eosinophilic granulomatosis with polyangiitis, eosinophilic pneumonia, and eosinophilic vasculitis.^8^^,^^9^ In the 2 cases reported above, patients also experienced persistent eosinophilia in the treatment of atopic dermatitis.

To our knowledge, these are the first reported cases of new onset, persistent, self-resolving hypereosinophilia with medication discontinuation. Once dupilumab was discontinued, there were no sequelae or other medical interventions, such as steroids or other medications as in previous studies. Of note, both patients in our cases are adult African American men with no history of asthma. The previously reported 5 cases of hypereosinophilia requiring discontinuation of dupilumab did not include demographic or medical history information.^7^

Olaguibel et al^9^ proposed that the etiology of dupilumab induced hypereosinophilia is the blocking of the IL-4 and IL-13 pathway. Blockade of the IL-4/IL-13 pathway reduces eosinophil migration and accumulation of blood by inhibiting eotaxin-3, VCAM-1, and TARC without simultaneously inhibiting eosinophilopoiesis in bone marrow.^9^

Dupilumab remains a safe mainstay of treatment for atopic dermatitis. In a 2023 efficacy study, 80.7% of respondents reported adequate disease control.^8^ This shows the effectiveness and sustained benefits of dupilumab. Of the 425 patients who completed the study, only 1% described their skin as “much worse.” Thus, it is abnormal for patients to significantly worsen on dupilumab, but if this occurs, hypereosinophilia should be considered.^8^ Laboratory assessment should be done to evaluate for hypereosinophilia and identify those with transient versus persistent abnormalities to avoid delay in appropriate management. Importantly, our 2 cases underwent extensive workup, however, eosinophilia resolved without sequelae over several months with discontinuation of dupilumab. Although the clinician may consider discontinuing dupilumab if the patient is not responding appropriately anyways without a workup, understanding the reason for medication failure is crucial. This allows for proper patient counseling and avoids unnecessary, invasive procedures. Lastly, we hope to spark further research on dupilumab induced hypereosinophilia.

## Conclusion

Eosinophilia in patients with atopic dermatitis on dupilumab remains a relatively new adverse side effect. Patients who fail to improve with dupilumab or similar medications should have lab work performed to assess for eosinophilia to appropriately distinguish those with transient eosinophilia and those that should be switched to another medication.

## Conflicts of interest

None disclosed.
